# Pathogen-targeting glycovesicles as a therapy for salmonellosis

**DOI:** 10.1038/s41467-019-12066-z

**Published:** 2019-09-06

**Authors:** Haibo Mu, Hu Bai, Feifei Sun, Yinyin Liu, Chunbo Lu, Yuanhao Qiu, Peng Chen, Yu Yang, Lili Kong, Jinyou Duan

**Affiliations:** Shaanxi Key Laboratory of Natural Products & Chemical Biology, College of Chemistry & Pharmacy, Northwest A&F University, 712100 Yangling, Shaanxi China

**Keywords:** Bacterial pathogenesis, Bacterial infection, Drug delivery

## Abstract

Antibiotic therapy is usually not recommended for salmonellosis, as it is associated with prolonged fecal carriage without reducing symptom duration or severity. Here we show that antibiotics encapsulated in hydrogen sulfide (H_2_S)-responsive glycovesicles may be potentially useful for the treatment of salmonellosis. The antibiotics are released in the presence of *Salmonella*, which is known to produce H_2_S. This approach prevents the quick absorption of antibiotics into the bloodstream, allows localized targeting of the pathogen in the gut, and alleviates disease symptoms in a mouse infection model. In addition, it reduces antibiotic-induced changes in the gut microbiota, and increases the abundance of potentially beneficial lactobacilli due to the release of prebiotic xylooligosaccharide analogs.

## Abstract (web summary)

Antibiotic therapy is usually not effective for salmonellosis. Here, the authors present an approach that may be useful for the treatment of salmonellosis, consisting of hydrogen sulfide (H_2_S)-responsive glycovesicles that release antibiotics in the presence of *Salmonella* in the gut.

## Introduction

As our primary interface with the external environment, the gastrointestinal tract represents a vast mucosal surface area vulnerable to attack by enteropathogens^1^. Salmonellosis, caused by food-borne *Salmonellae* is one of the most frequently occurring intestinal diseases. Globally, there are ~153 million cases of gastroenteritis and 57,000 deaths each year^2^.

The typical symptoms of salmonellosis, involving stomach cramps, nausea, and acute diarrhea, appear ~6–72 h after consumption of contaminated food or water^3^. This illness usually lasts 4–7 days, and most people recover without treatment. Current recommendations are to treat most patients with this illness with oral rehydration therapy but not with antimicrobial agents^4,5^. Antimicrobial therapy should be considered for patients who are severely ill (for example, those with severe diarrhea, high fever, or manifestations of extraintestinal infection) and for people at increased risk of invasive disease (infants, older adults, and the debilitated or immunosuppressed^6,7^.

Antimicrobial therapy of salmonellosis is known to be associated with prolonged fecal carriage, without reducing symptom duration or severity, and may even increase the rates of long-term shedding of pathogens^8^. Systemically absorbed antibiotics such as quinolones and sulfonamides are readily taken up into the bloodstream if given orally, which makes them difficult to retain high concentrations at the site of enteric infections^9,10^. Poorly absorbed oral antibiotics such as aminoglycosides and β-lactam families allow localized enteric targeting of pathogens^11^. However, these antibiotics alter the balance of gut flora, leading to a possible overgrowth of opportunistic pathogenic bacteria^12^.

To overcome the inherent defects of antimicrobial therapy in patients with salmonellosis, here we introduce a versatile vesicle-based system for delivering antibiotics to target the illness-causative agent, *Salmonella enterica serovar Typhimurium* selectively. This study might open up an avenue to develop pathogen-targeting antimicrobial glycovesicles to resolve enteric infections with a minimal risk of adverse outcomes.

## Results

### Design of pathogen-targeting antimicrobial glycovesicles

Hydrogen sulfide (H_2_S) production is considered a typical characteristic of *Salmonella* and an important marker for *Salmonella* isolation. *Salmonella* produce H_2_S from L-cysteine, by the activity of cysteine desulfhydrase^13^ or produces H_2_S from thiosulfate by thiosulfate reductase^14^. Colonies that produce H_2_S are considered as the most clinical relevant and significant^13^.

A H_2_S-cleavable amphiphilic molecule (AM) is constructed by conjugation of xyloligosaccharide analogs with 1-dodecanethiol through a disulfide bond bridge (Fig. 1a and Supplementary Figs. 1–14). The spontaneous self-assembly of AMs forms spherical vesicles (Fig. 1b) with an average diameter of 100 nm (Fig. 1c), which elicit a clear Tyndall effect (Fig. 1b inset, right). The critical aggregation concentration of AM in water is calculated to be 0.0225 mg mL^−1^ (Supplementary Fig. 16).

**Fig. 1:**
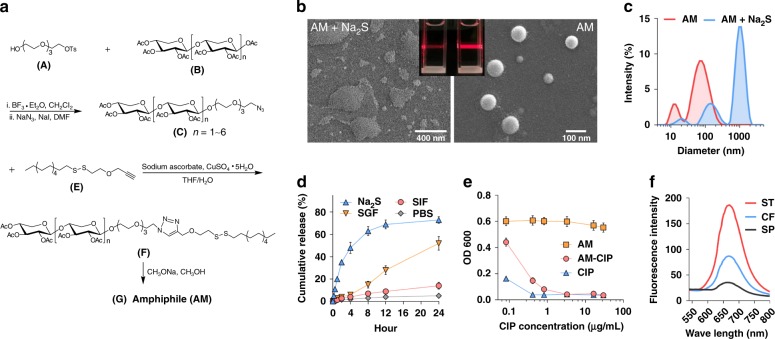
Synthesis and characterization of AM vesicles. **a** Synthetic route of amphiphile (AM). **b** Scanning electron microscopic (SEM) images of AM vesicles with (left) or without (right) Na_2_S (100 μM). Scale bars represent 400 nm (left) and 100 nm (right), respectively. Inset: Tyndall effect of AM vesicles with or without Na_2_S. **c** Hydrodynamic size distribution from DLS analysis in the absence or presence of Na_2_S (100 μM). **d** Cumulative release profiles of AM-CIP in PBS, PBS with Na_2_S (100 μM), simulated gastric fluid (SGF), or simulated intestinal fluid (SIF). Each is performed in triplicate. **e** In vitro bactericidal activity of free CIP (CIP), CIP-loaded AM vesicles (AM-CIP), and blank AM vesicles against *S. typhimurium* bacteria. Each is performed in triplicate. **f** Fluorescence intensity of Fluo-loaded AM vesicles (containing 10 μM Fluo) incubated with *S. typhimurium* (ST), *C. freudii* (CF), or *S. paratyphi* A (SP) (10^6^ CFU mL^−1^) for 12 h. Data are means ± SD, representative of three technical repeats. Source data are provided as a Source Data file

The spherical vesicles above collapse in the presence of 100 μM sodium sulfide (Na_2_S) (Fig. 1b), accompanied with an increasing diameter (Fig. 1c) and a disappeared Tyndall effect (Fig. 1b inset, left). These data results imply that Na_2_S might result in the disassembly of AM vesicles, which is probably due to the decomposition of amphiphilic molecule caused by the reduction of disulfide bond.

The drug release behavior of AM vesicles was investigated using the fluoroquinolone antibiotic, ciprofloxacin hydrochloride (CIP) as a model drug, since fluoroquinolones are considered first-line treatment in salmonellosis^15^. From UV/Vis absorption spectra, the CIP loading efficiency is 9.2% (w/w), indicating a good drug-loading capability of AM vesicles. There are only about 2%, 4%, and 6% CIP released, respectively, from CIP-loaded AM vesicles (AM-CIP) when incubated 4 h in PBS, simulated gastric fluid (SGF, pH 2) and simulated intestinal fluid (SIF, pH 7) (Fig. 1d). Since the mean gastric emptying time of adults is less than 4 h^16^, this finding indicates AM-CIP is fairly stable in gastrointestinal environment when given orally. In contrast, CIP is released from AM-CIP quickly and the released CIP reaches to ~50% at 4 h later in the presence of Na_2_S (100 μM), which is consistent with the previous observation that Na_2_S induces the disassembly of AM vesicles. Indeed, Na_2_S induces CIP release from AM-CIP in a dose-dependent manner (Supplementary Fig. 17).

To see whether pathogens can decompose AM vesicles, the H_2_S-producing pathogen, *S. typhimurium* is co-cultured with AM-CIP. AM-CIP at low concentrations (<3.28 μg mL^−1^) has a weaker bactericidal capacity than the same amount of CIP does, while their activities are comparable at high concentrations (Fig. 1e). This data impliy that *S. typhimurium* can produce sufficient H_2_S to decompose AM-CIP, which enables the direct contact between the released CIP and pathogens. The H_2_S concentration produced by *S. typhimurium* (10^6^ CFU mL^−1^) is determined to be 125 μM, according to the colorimetric method^17^.

An H_2_S-responsive fluorescence probe, Fluo (Supplementary Fig. 15) is also employed to evaluate whether the disassembly of AM vesicles is dependent on specific bacteria. There are far stronger fluorescence intensity in the Fluo-loaded AM vesicles when incubated with *S. typhimurium* (producing H_2_S) than that of *S. paratyphi* A (not producing H_2_S) (Fig. 1f), an observation that AM vesicles can only be decomposed by H_2_S-producing organisms such as *S. typhimurium* selectively. Although other H_2_S-producing organisms such as *C. freudii* also induces mild fluorescence at the same cell density as *S. typhimurium* (Fig. 1f), the high abundance of *S. typhimurium* in infected intestines will facilitate the disassembly of AM vesicles by this pathogenic bacteria.

### Pathogen-targeting glycovesicles treat salmonellosis efficiently

**Fig. 2:**
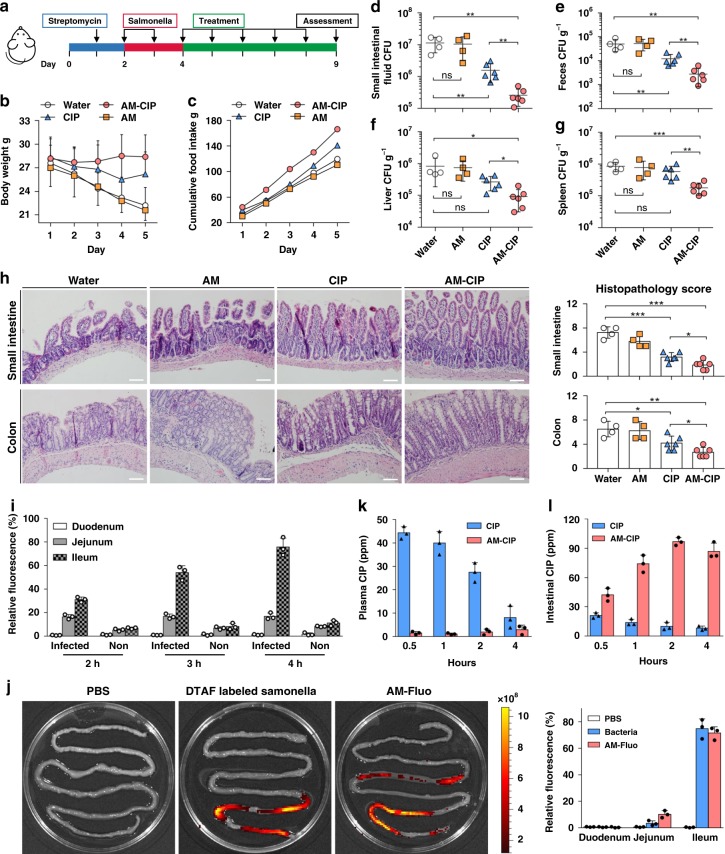
Therapeutic efficacy in acute intestinal infection model. **a** The study protocol including streptomycin pretreatment and *S. typhimurium* inoculation on mice, followed by the treatments [AM (100 mg kg^−1^), CIP (10 mg kg^−1^), or AM-CIP (110 mg kg^−1^) daily]. **b** Body weight. *n* = 4–6 mice per group. **c** Cumulative food intake. *n* = 4–6 mice per group. Quantification of bacterial burden in the small intestine (**d**), feces (**e**), liver (**f**), and spleen (**g**) of infected mice with different treatments. **h** H&E stained sections of intestine tissues from infected mice after with individual treatments. Scale bar, 100 µm. Histogram represents combined histopathology score (*n* = 4–6 mice per group). **i** Fluorescent intensity of small intestines from normal or infected mice 2 h, 3 h, and 4 h after administration of Fluo-loaded AM vesicles (*n* = 3 mice per group). **j** Fluorescence images of small intestines from infected mice 12 h after administration of PBS, 5-DTAF-labeled *S. typhimurium* or Fluo-loaded AM vesicles (*n* = 3 mice per group). **k**, **l** Mean plasma or intestinal concentration of CIP following a single oral administration of AM-CIP (110 mg kg^−1^) or CIP (10 mg kg^−1^) in normal mice (*n* = 3 mice per group). Data are shown as mean ± SD, and each dot represents one animal. A single asterisk indicate p-values of <0.05, double asterisks indicate *p*-values of <0.01, triple asterisks indicate *p*-values of <0.001, *ns* no statistical significance (two-tailed Student’s *t*-test). Source data are provided as a Source Data file

An acute intestinal infection mouse model is developed using *Salmonella enterica serovar Typhimurium* (Fig. 2a). *Salmonella* infections result in a constant decline of body weight (Fig. 2b). CIP treatment can partially prevent weight loss and increase food uptake, while AM-CIP has a more pronounced effect than CIP does (Fig. 2b, c). CIP therapy leads to a decrease of *Salmonella* counts in the gastrointestinal tract (Fig. 2d, e), but it doesn’t affect pathogen burden at the extraintestinal sites such as liver and spleen at the tested doses (Fig. 2f, g). In contrast, AM-CIP can efficiently reduce *S. typhimurium* infections in the gastrointestinal tract and thereby alleviate its dissemination into liver and spleen (Fig. 2d–g). In line with the above observation, severe tissue destruction and inflammatory infiltrates are observed in enteric mucosa and submucosa of small intestines and colons from untreated *S. typhimurium* infected mice (Fig. 2h). These epithelial damage and inflammation are greatly alleviated after CIP-AM treatment, which is superior to that of CIP.

To find out drug release behavior in the intestine, infected or normal mice are orally administrated with Fluo-loaded AM vesicles. The ileum from infected mice, but not from normal ones elicits the strongest fluorescence intensity in a time-dependent manner (Fig. 2i), implying that 1) *S. typhimurium* triggers the release of the probe from AM vesicles and 2) there is higher relative abundance of *S. typhimurium* in ileum. Further, infected mice are administrated with fluorescence (5-DTAF)-labelled *S. typhimurium* or Fluo-loaded AM vesicles. The fluorescence-labelled bacteria mainly retain in ileum (Fig. 2j), even after 12 h post oral administration, an observation that the ileum is one primary site of *Salmonella* infection. Consistently, there is strongest fluorescence intensity in ileum after administration of Fluo-loaded AM vesicles (Fig. 2j). Taken together, these findings indicate that AM vesicles are disassembled on-demand at the site of infection.

Unlike poorly absorbed oral antibiotics, absorbable antibiotics such as the fluoroquinolone family cannot retain therapeutic concentrations at the site of enteric infection^10,11^. As expected, CIP is readily absorbed and taken into the bloodstream in 30 min after oral administration. Differently, there are a little amount of CIP detected in the blood and most CIP remains in the intestine after 4 h if orally given with AM-CIP (Fig. 2k, l). This data indicate that AM vesicles can prevent the quick absorption of systematically available antibiotics into the bloodstream.

### Glycovesicles reduce antibiotic-induced changes in gut microbiota

One common side effect of antibiotic treatment for enteric infections is collateral damage to the gut microbiota composition, resulting in a possible overgrowth of opportunistic pathogenic bacteria. Now it is believed that a repeated exposure to therapeutic doses of antimicrobials can even lead to long-lasting disruption of the gut flora and this side effect is not restricted to orally applied antibiotics^18,19^.

**Fig. 3:**
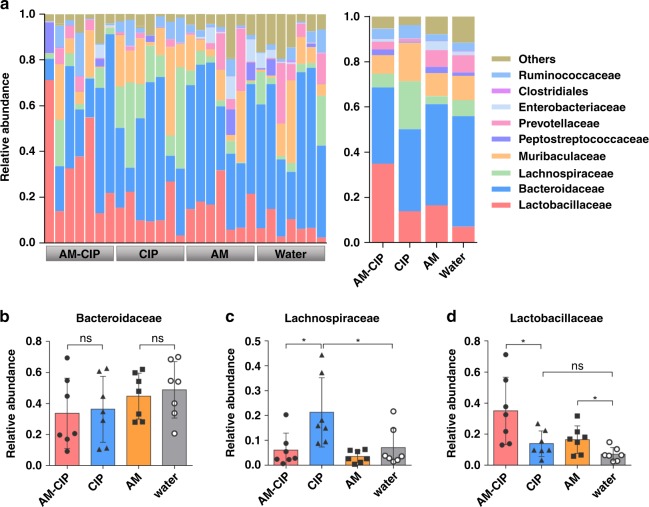
Gut microbial analysis in infected mice after treatment. **a** Left: bacterial taxonomic profiling at family level of the gut microbiota in feces from infected mice post therapy. Right: average composition of the family level in four groups. Relative abundance of *Bacteroidaceae* (**b**), *Lachnospiraceae* (**c**), and *Lactobacillaceae* (**d**) obtained in fecal microbiota from the LefSe results. Data are shown as mean ± SD, *n* = 7 mice per group and each dot represents data from an individual animal. A single asterisk indicate *p*-values of <0.05, ns, no statistical significance (two-tailed Student’s *t*-test). Source data are provided as a Source Data file

To assess the effect of AM-CIP on gut microbial communities, we perform a pyrosequencing-based analysis of bacterial 16 S rRNA in caecal feces from *S. typhimurium* infected mice treated with water, AM, CIP, or AM-CIP (Fig. 3a). Similar abundance of the *Bacteroidaceae* family is observed in all groups (Fig. 3b). One most striking different is that CIP treatment induces an overgrowth of the *Lachnospiraceae* family (Fig. 3c). Several members from this family are considered opportunistic pathogens, which have been identified in inflamed samples such as diabetic wounds^20^, irritable bowel syndrome^21,22^, subgingival crevice^23^, and cystic fibrosis^24^. The other difference is the variance of abundance of the *Lactobacillaceae* family for each treatment. Both AM-CIP and AM treatment, but not CIP greatly increase abundance of the *Lactobacillaceae* family (Fig. 3d), which is probably due to the prebiotic xylooligosaccharide analogs released after collapse of AM vesicles. This is encouraging since several strains from the *Lactobacillaceae* family are shown to be highly antagonistic to *Salmonella* pathogens and protect against *Salmonella* infections in the gastrointestinal tract^25–27^. It is reasonable that AM treatment does not increase *Lactobacillaceae* as much as AM-CIP treatment does, due to the suppressive effect of abundant *Salmonella* pathogens on *Lactobacillaceae* in AM-treated groups. To see whether AM-CIP shaped gut microbiota is beneficial to the host, we transfer the gut microbiota from AM-CIP or CIP-treated *Salmonella*-infected donor mice to infected recipient mice. The recipient mice received with AM-CIP-shaped gut microbiota have lower pathogen burden and tissue inflammation than that in mice received with CIP-shaped one (Supplementary Fig. 18).

**Fig. 4:**
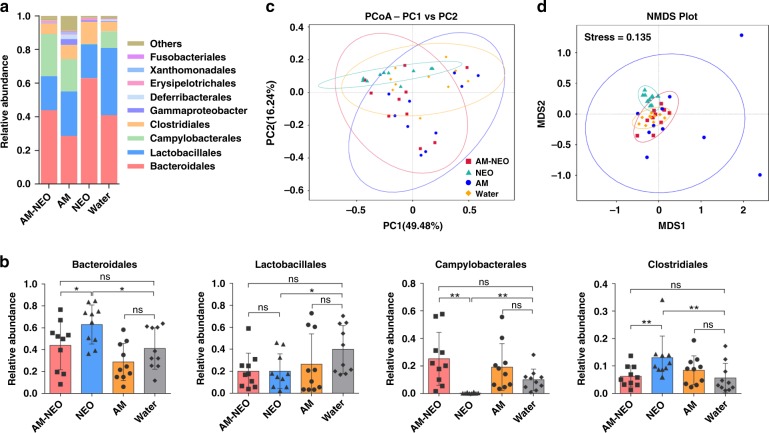
AM vesicle cloak alleviates neomycin induced damages to the gut microbiota in normal mice. **a** Average bacterial taxonomic profiling at order level of the gut microbiota in four groups. **b** Relative abundance of the bacterial order obtained in fecal microbiota from the LefSe results. Data are shown as mean ± SD, *n* = 10 mice per group and each dot represents data from an individual animal. A single asterisk indicate *p*-values of <0.05, double asterisks indicate *p*-values of <0.01, ns, no statistical significance (two-tailed Student’s *t*-test). Source data are provided as a Source Data file. **c** Nonmetric multidimensional scaling score plot based on Bray-Curtis. **d** UniFrac-based PCoA score plot based on weights

To further define whether this strategy can minimize antibiotic-induced collateral damage to gut homeostasis, one poorly absorbed antibiotic, neomycin (NEO) is encapsulated to yield neomycin-loaded AM vesicles (AM-NEO). Although total NEO content in the intestine is quite similar whatever NEO or AM-NEO is given (Supplementary Fig. 19), as expected, NEO treatment significantly increased the relative abundance of *Bacteroidales* and *Clostridiales* and decreased content of *Lactobacillales* and *Campylobacterales*. In contrast, AM-NEO treatment has not those effects (Fig. 4a, b). The microbiota structural changes were then analyzed using unsupervised multivariate statistical methods including UniFrac distance-based principal coordinate analysis (PCoA) and nonmetric multidimensional scaling (NMDS) (Fig. 4c, d). NEO treatment presented a centralized clustering of microbiota composition, indicating NEO reduces microbiota diversity. On the contrary, AM-NEO treatment does not alter gut microbiota composition as NEO treatment does.

## Discussion

Although antibiotics are a life-saving tool for the treatment of bacterial infections, antimicrobial therapy is not routinely recommended for patients with enteric infections^8^. Antibiotic use will induce important collateral damage to host-associated microbial communities^28,29^. This puzzle prompts us to develop a strategy to eliminate enteric pathogens with minimized negative influence on gut microbiota during antimicrobial therapy.

Disulfide bonds have been widely used to develop reduction-responsive drug-delivery systems for cancer therapy, since it can be rapidly degraded and selectively release cargoes in tumor tissues, which contain higher glutathione levels than that in normal tissues^30^. To our interest, the disulfide bond can also be easily broken down by H_2_S, a Janus-faced molecule which can be produced selectively by several sulfate-reducing bacteria such as *Streptococcus*, *Fusobacterium*, *Salmonella*, *Enterobacter*, and *Helicobacter* in the gastrointestinal tract^31–33^.

In this study, xylo-oligosaccharides are conjugated with a long-chain fatty chain by a disulfide bond bridge to form an amphiphilic molecule, whose self-assembly can generate an H_2_S-responsive antibiotic delivery system. In vivo toxicity evaluation indicates that this drug-delivery system is biocompatible and safe during oral administration in mouse models (Supplementary Fig. 20).

**Fig. 5:**
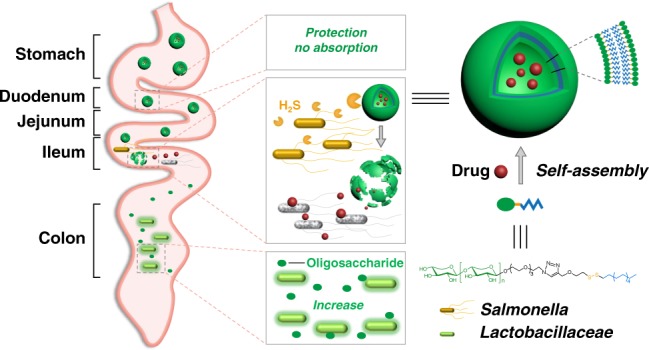
A proposed model for how AM vesicles resolve salmonellosis efficiently. The envisioned paradigm of AM vesicles to protect ciprofloxacin (CIP) from absorption, eliminate *Salmonella* bacteria locally and promote *Lactobacillaceae* growth

By using a well-established acute *Salmonella* infection model, we find that this system can prevent the quick absorption of ciprofloxacin in the small intestine and release the antibiotic at *Salmonella*-rich infectious sites. The releasing antibiotic is uptaken by *Salmonella* pathogens nearby and thus dramatically decrease bacterial burden. After cleavage of the disulfide bond, the generated xylooligosaccharide analogs can promote the growth of the beneficial microbiota, *Lactobacillaceae* in the gastrointestinal tract (Fig. 5). It is known that oligosaccharide prebiotics such as xylooligosaccharide could only be utilized by a limited number of beneficial bacteria including lactobacilli and selectively proliferate these organisms^34^.

Most commercially available antibiotics including ciprofloxacin are easily absorbed and are rapidly taken up into the bloodstream, which make them unfavorable for localized enteric targeting of pathogens in the gastrointestinal tract^9,10,35^. This drug-delivery system retrieves absorbable antibiotics above as a drug reservoir to treat enteric infections caused by H_2_S-producing pathogens, which might be extremely important in case of experiencing highly virulent antibiotic-resistant infections in the gastrointestinal tract.

This drug-delivery system is further employed to load one poorly absorbed antibiotic to find out whether it can ameliorate antibiotic-induced collateral damage to the gut microbiota composition. It is intriguing that this delivery system does not disturb the microbiota greatly as the poorly absorbed antibiotic alone does (Fig. 4).

In summary, we develop an H_2_S-responsive antibiotic delivery system for selective targeting of *Salmonella*, the salmonellosis-causative pathogen. This system includes the following features: (1) prevent quick absorption of oral antibiotics into the bloodstream; (2) allow antibiotics to target enteric pathogens locally and thereby alleviates disease symptoms; (3) ameliorate antibiotic-induced damage to gut microbiota greatly; and (4) increase beneficial microbiota *Lactobacillaceae* by the releasable prebiotic xylooligosaccharide analogs. This study might open up an avenue to develop pathogen-targeting antimicrobial glycovesicles to resolve enteric infections.

## Methods

### Chemical reagents

Tetrathylene glycol (99%), Tosyl chloride (99%), Sodium acetate (≥99%), Acetic anhydride (≥98.5%), Sodium azide (99.5%), Pyridine (99%) were obtained from Aladdin Industrial Inc. (Shanghai, China). Xylo-oligosaccharides (95%) was purchased from Shanghai Yuanye Biological Technology Co., Ltd. (Shanghai, China). Dodecane-1-thiol (≥95%), 2-mercaptoethanol (99%) were bought from TCI (Shanghai, China). Propargyl bromide (99%) was obtained from Energy Chemical Reagent Co. All other reagents were of analytical grade and used as received. NMR spectra were recorded on a Bruker 500 MHz Spectrometer with working frequencies of 500 MHz for ^1^H and 125 MHz for ^13^C, respectively, in CDCl_3_ or CD_3_OD. The residual signals from CDCl_3_ (^1^H: δ 7.26 ppm; ^13^C: δ 77.00 ppm) or CD_3_OD (^1^H: δ 3.31 ppm; ^13^C: δ 49 ppm) were used as internal standards. HRMS were acquired using a high resolution mass spectrometer (New ultrafleXtreme; Bruker Daltonik, Bremen, Germany). Ions were generated with a pulsed 337 nm nitrogen laser and accelerated to 25 kV. All spectra were obtained in the reflectron mode with delayed extraction of 200 ns. For sample preparation, 0.5 μL of 2,5-dihydroxybenzoic acid (DHB) (10 mg mL^−1^) in 30% ethanol was spotted onto a target plate (MTP 384 target plate ground steel, Bruker Daltonik). After dried, an aliquot (0.5 μL) of the sample solution was spotted onto the DHB crystal and dried. All HRMS spectra were obtained from Na^+^ adduct ions.

### Syntheses of AM

The synthetic details and characterization of compound A–G can be found in Supplementary Note 1 and Supplementary Figs. 1–14. The synthesis and characterization of Fluo were shown in Supplementary Fig. 15.

### The preparation, characterization, and stability of the vesicles

Three milligram of amphiphile (AM) were dissolved in 1 mL methanol, and then the organic solvent was evaporated to form a dried lipid film. The lipid film was rehydrated with 3 mL of deionized water, or 1 mg rhodamine B (RhB), or 3 mg ciprofloxacin (CIP), followed by vortexing for 1 min and sonicating for 30 min to produce AM-CIP vesicles, purified by dialysis (molecular weight cutoff 3500 Da) in deionized water. The unencapsulated CIP in the dialysate was quantified by UV-Vis at 277 nm. For preparation of probe-loaded vesicles, 0.2 mg of Fluo was dissolved in methanol with 3 mg of AM before evaporation.

### Release behaviors

The kinetics of ciprofloxacin release was studied from the prepared AM-CIP. The fresh prepared AM-CIP solution (2 mg mL^−1^) was initially incubated with or without Na_2_S in PBS (SIP or SGF) at 37 °C. At predetermined time points, released CIP was separated by filtration using 10 kDa MWCO Amicon centrifugal filters (EMD Millipore, Billerica, CA, USA). CIP concentration was determined by measuring the absorbance at 277 nm.

### Bacteria strains

*Salmonella enterica serovar typhimurium* SL1344, *Salmonella paratyphi* A ATCC9150, and *Citrobacter freundii* ATCC43864 were purchased from BeNa Culture Collection (Beijing, China). The bacteria strains were routinely cultured in Luria-Bertani (LB) broth at 37 °C with moderate reciprocal shaking overnight for future use.

### In vitro bactericidal activity

Bacteria samples (10^6^ CFU mL^−1^) were mixed well with LB including different concentrations of CIP (0.082–29.820 μg mL^−1^), equivalent AM-CIP or AM. After incubation at 37 °C for 24 h, the OD_600_ was monitored.

### Mouse strains and husbandry

Kunming mice were obtained from Xi’an Jiaotong University. Female mice (5 weeks old) were housed in cages containing sawdust bedding in holding rooms with a temperature 25 °C and a relative humidity of about 40%. Deionized water and food were provided ad libitum. All animal procedures complied with all relevant ethical regulations and were approved by the Northwest A&F University Animal Care Committee (NWAFU-314020038). Mice were given 2 week to acclimate before experimentation.

### Mouse infections

As described^8^, water and food were withdrawn 4 h before per os treatment with 20 mg of streptomycin. Afterward, animals were supplied with water and food ad libitum. At 20 h after streptomycin treatment, water and food were withdrawn again for 4 h before the mice were infected via oral gavage with 10^9^ CFU of *S. typhimurium* in 100 μL PBS. Thereafter, drinking water food ad libitum was offered immediately. One day later, mice received oral gavage with water, AM (100 mg kg^−1^), CIP (10 mg kg^−1^), or AM-CIP (110 mg kg^−1^) once every day for 5 days.

### *S. typhimurium* burden in tissues

After collection of fresh fecal pellets, animals were euthanized by cervical dislocation. The intestine, spleen, and liver were collected in sterile PBS. Homogenates were then serially diluted and plated onto LB agar to enumerate colony-forming units (CFU).

### Gut microbiota profiling

Fresh fecal pellets were collected, stored in liquid nitrogen, transported to Novogene (Beijing, China), packed with dry ice, and then immediately stored in a −80 °C refrigerator before extraction of total DNA. The 16 S rRNA gene comprising V3–V4 regions was amplified using common primer pair and the microbial diversity analysis was performed as described^36^. Briefly, the raw sequences were first quality-controlled using QIIME with default parameters, then demultiplexed and clustered into species-level (97% similarity) operational taxonomic units (OTUs). OUT generation is based on GreenGene’s database and the reference-based method with SortMeRNA. Strain composition analysis, alpha diversity analysis and beta diversity analysis were performed using QIIME. Discriminative taxa were determined using LEfSe (LDA Effect Size).

### Fecal transplantation

Fecal transplant was performed as a reported protocol^37^. Briefly, 8-week-old female donor mice (*n* = 5 mice per group) were infected and treated with CIP or AM-CIP for 5 days as previous. Then stools were collected daily for the subsequent 6 days under a laminar flow hood in sterile conditions. Stools from donor mice of each treated group were pooled and 100 mg was resuspended in 1 mL of sterile PBS with vigorous mixing, and then subjected to centrifugation at 600 g for 5 min. The supernatant was collected and used as transplant material. Fresh transplant material was prepared on the same day of transplantation within 10 min before oral gavage to prevent changes in bacterial composition. Eight-week-old female recipient mice (*n* = 9–10) were infected beforehand and inoculated daily with fresh transplant material (200 μL for each mouse) by oral gavage for 6 days, before being killed for subsequent analysis.

### Blood and intestine drug concentration

As previously described^38^, 7-week-old uninfected Kunming female mice were randomly divided into two groups (*n* = 12). All experimental animals received intragastrically a single dose (300 μL) of CIP or AM-CIP. After treatments, three mice per group were humanly sacrificed at each time point post-administration 0.5, 1, 2, and 4 h. Blood samples (800 μL) were collected into heparinized tubes. The samples were centrifuged for 15 min (4 °C, 2000 rpm), and the plasma was separated and transferred into polypropylene tubes. The small intestines were also collected and flushed with PBS. The ciprofloxacin concentrations in blood and intestine were then determined by ELISA kit (ZIKER Biological Technology, Shenzhen, China).

### In vivo intestinal tract site-specific response

Seven-week-old infected Kunming female mice were gavaged with 300 μL of AM-Fluo (*n* = 3 mice per group). Intestinal tracts including duodenum, jejunum, and ileum from each mouse were collected at indicated time points (2, 3, 4 h) after administration. The tissues were rinsed inside with DMSO, and the consequent DMSO was subjected to fluorescence determination using a fluorescence spectrophotometer, Ex/Em = 520/670 nm. For in vivo images, *S. typhimurium* were labeled using the dye 5-(4–6-dichlorotriazinyl) aminofluorescein (5-DTAF)^39^. Infected mice (*n* = 3 per group) were orally gavaged with 200 μL suspension of 5-DTAF-labeled bacteria (10^9^ CFU) containing 0.2 M NaHCO_3_, or administered orally with 200 μL suspension of AM-Fluo, PBS as a control. At 12 h after administration, the intestinal tracts were dissected, rinsed with PBS, and then imaged using an in vivo imaging system (IVIS, Lumina XRMS III, PerkinElmer). Fluorescence intensity of images were calculated using Living Image software from Caliper Life Sciences.

### In vivo toxicity study

To evaluate the acute toxicity of the AM-CIP in vivo, 7-week-old uninfected Kunming female mice were orally administered with AM-CIP once daily for 5 consecutive days. Mice administered with deionized water were tested in parallel as a negative control. During the experimental period, the mouse body weight and food intake were monitored by weighing the mice daily. On day 6, mice were killed and sections of the small and large intestine tissues were processed for histological examination. The small and large intestines were cut to small sections as duodenum, jejunum, ileum, and colon and rinsed inside with PBS to remove internal residues. The longitudinal tissue sections were fixed in neutral-buffered 10% formalin for 15 h, transferred into 70% ethanol, and embedded in paraffin. The tissue sections were cut with 5 μm thickness and stained with H&E assay.

### Histopathology scoring

Histopathology scoring was performed on H&E stained small intestine (ileum) and colon tissue sections according to the criteria below: (a) Integrity of the intestinal epithelium: intact and no pathological changes (0), mild (1), moderate (2), and severe destruction (3); (b) Mucosal inflammation: no inflammatory infiltrates (0), rare (1), moderate (2), and massive invasion of immune cells (3); (c) Submucosa: no pathological changes (0), rare (1), moderate (2), or massive invasion of immune cells and edema (3).

### Statistical analyses

Statistical analysis and graphing were done with Graphpad Prism. Quantitative results are presented as mean values with standard deviation (SD). The two-tailed Student’s *t*-test was used to compare two experimental groups. *P* < 0.05 was considered statistically significant.

### Reporting summary

Further information on research design is available in the Nature Research Reporting Summary linked to this article.

## Supplementary information

Supplementary Information

Peer Review File

Reporting Summary

Source Data

## Data Availability

All the sequence data generated for this study have been deposited in NCBI Sequence Read Archive (SRA) under accession number PRJNA558567 and PRJNA557572. The source data underlying Figs. 1c–f, 2b–1, 3a–d, 4a–b and Supplementary Figs. 16–20 are provided as a Source Data file. All data supporting the findings of this study are present in the article and Supplementary Information, or are available from the corresponding author upon reasonable request.

## References

[1] Turner JR (2009). Intestinal mucosal barrier function in health and disease. Nat. Rev. Immunol..

[2] Hunter, J. C. & Watkins, L. K. F. *CDC Yellow Book 2018: Health Information for International Travel*. (ed. Brunette, G. W) Ch. 3, 304–305 (Oxford University Press, New York, USA, 2017).

[3] Gordon D, Victoria J, Sophie P, Pietro M (2015). Immunity to salmonellosis. Immunol. Rev..

[4] Riddle MS, DuPont HL, Connor BA (2016). ACG clinical guideline: diagnosis, treatment, and prevention of acute diarrheal infections in adults. Am. J. Gastroenterol..

[5] Cameron D (2017). Probiotics for gastrointestinal disorders: proposed recommendations for children of the Asia-Pacific region. World J. Gastroenterol..

[6] Zollner-Schwetz I, Krause R (2015). Therapy of acute gastroenteritis: role of antibiotics. Clin. Microbiol. Infect..

[7] Chen H-M, Wang Y, Su L-H, Chiu C-H (2013). Nontyphoid Salmonella infection: microbiology, clinical features, and antimicrobial therapy. Pediatr. Neonatol..

[8] Endt K (2012). Peroral ciprofloxacin therapy impairs the generation of a protective immune response in a mouse model for Salmonella enterica serovar Typhimurium diarrhea, while parenteral ceftriaxone therapy does not. Antimicrob. agents Chemother..

[9] Fish DN, Chow AT (1997). The clinical pharmacokinetics of levofloxacin. Clin. Pharmacokinet..

[10] Marzo A, Dal Bo L (1998). Chromatography as an analytical tool for selected antibiotic classes: a reappraisal addressed to pharmacokinetic applications. J. Chromatogr. A.

[11] Taylor DN (2005). Poorly absorbed antibiotics for the treatment of Traveler’ diarrhea. Clin. Infect. Dis..

[12] Bäumler AJ, Sperandio V (2016). Interactions between the microbiota and pathogenic bacteria in the gut. Nature.

[13] Lin D, Yan M, Lin S, Chen S (2014). Increasing prevalence of hydrogen sulfide negative Salmonella in retail meats. Food Microbiol..

[14] Burns JL, DiChristina TJ (2009). Anaerobic respiration of elemental sulfur and thiosulfate by Shewanella oneidensis MR-1 requires psrA, a homolog of the phsA gene of Salmonella enterica Serovar Typhimurium LT2. Appl. Environ. Microbiol..

[15] Crump JA, Barrett TJ, Nelson JT, Angulo FJ (2003). Reevaluating fluoroquinolone breakpoints for Salmonella enterica Serotype Typhi and for non-Typhi Salmonellae. Clin. Infect. Dis..

[16] Kuo B (2008). Comparison of gastric emptying of a nondigestible capsule to a radio-labelled meal in healthy and gastroparetic subjects. Aliment. Pharmacol. Ther..

[17] Sun W (2013). A two-photon fluorescent probe with near-infrared emission for hydrogen sulfide imaging in biosystems. Chem. Commun..

[18] Lankelma JM (2017). Antibiotic-induced gut microbiota disruption during human endotoxemia: a randomised controlled study. Gut.

[19] Palleja A (2018). Recovery of gut microbiota of healthy adults following antibiotic exposure. Nat. Microbiol..

[20] Grice EA (2010). Longitudinal shift in diabetic wound microbiota correlates with prolonged skin defense response. Proc. Natl Acad. Sci. USA.

[21] Kassinen A (2007). The fecal microbiota of irritable bowel syndrome patients differs significantly from that of healthy subjects. Gastroenterology.

[22] Li E (2012). Inflammatory bowel diseases phenotype, C. difficile and NOD2 genotype are associated with shifts in human ileum associated microbial composition. PLoS One.

[23] Kroes I, Lepp PW, Relman DA (1999). Bacterial diversity within the human subgingival crevice. Proc. Natl Acad. Sci. USA.

[24] van der Gast CJ (2010). Partitioning core and satellite taxa from within cystic fibrosis lung bacterial communities. Isme J..

[25] Castillo NA, Perdigón G, de Moreno de LeBlanc A (2011). Oral administration of a probiotic Lactobacillus modulates cytokine production and TLR expression improving the immune response against Salmonella enterica serovar Typhimurium infection in mice. BMC Microbiol..

[26] Casey PG (2007). A five-strain probiotic combination reduces pathogen shedding and alleviates disease signs in pigs challenged with Salmonella enterica Serovar Typhimurium. Appl. Environ. Microbiol..

[27] Feng J, Wang L, Zhou L, Yang X, Zhao X (2016). Using in vitro immunomodulatory properties of lactic acid bacteria for selection of probiotics against Salmonella infection in broiler chicks. PLoS One.

[28] Sana TG, Monack DM (2016). The dark side of antibiotics. Nature.

[29] Wypych TP, Marsland BJ (2018). Antibiotics as instigators of microbial dysbiosis: implications for asthma and allergy. Trends Immunol..

[30] Aoyama E, Fuchida H, Oshikawa Y, Uchinomiya S, Ojida A (2016). Intracellular delivery of chemical probes using a glutathione-responsive traceless tag. Chem. Commun..

[31] Ito S, Nagamune H, Tamura H, Yoshida Y (2008). Identification and molecular analysis of βC–S lyase producing hydrogen sulfide in Streptococcus intermedius. J. Med. Microbiol..

[32] Lee H, Kho H-S, Chung J-W, Chung S-C, Kim Y-K (2006). Volatile sulfur compounds produced by helicobacter pylori. J. Clin. Gastroenterol..

[33] Basic A, Blomqvist M, Dahlén G, Svensäter G (2017). The proteins of Fusobacterium spp. involved in hydrogen sulfide production from L-cysteine. BMC Microbiol..

[34] De Maesschalck C (2015). Effects of Xylo-oligosaccharides on broiler chicken performance and microbiota. Appl. Environ. Microbiol..

[35] Scarpignato C, Pelosini I (2005). Rifaximin, a poorly absorbed antibiotic: pharmacology and clinical potential. Chemotherapy.

[36] Qin J (2012). A metagenome-wide association study of gut microbiota in type 2 diabetes. Nature.

[37] Chang C-J (2015). Ganoderma lucidum reduces obesity in mice by modulating the composition of the gut microbiota. Nat. Commun..

[38] Breda SA (2013). Systemic exposure, tissue distribution, and disease evolution of a high solubility ciprofloxacin–aluminum complex in a murine model of septicemia induced by Salmonella enterica Serotype Enteritidis. Mol. Pharm..

[39] Mu H (2016). Chitosan conjugation enables intracellular bacteria susceptible to aminoglycoside antibiotic. Glycobiology.

