# Supplementary material for: Pathogen-targeting glycovesicles as a therapy for salmonellosis
Source: Nat Commun. 2019 Sep 6;10:4039. doi: 10.1038/s41467-019-12066-z (PMC6731243; doi:10.1038/s41467-019-12066-z)
Supplement: Supplementary file 1 — Supplementary Information [file 41467_2019_12066_MOESM1_ESM.pdf]

Supplementary information

Pathogen-Inspired Antimicrobial Glycovesicles to Resolve Salmonellosis

Haibo Mu[1], Hu Bai[1], Feifei Sun, Yinyin Liu, Chunbo Lu, Yuanhao Qiu, Peng Chen, Yu Yang, Lili Kong and Jinyou Duan*

Shaanxi Key Laboratory of Natural Products & Chemical Biology, College of Chemistry & Pharmacy, Northwest A&F University, Yangling 712100, Shaanxi, China

[1] These author contributed equally to this work

* Correspondence

Jinyou Duan, PhD

E-mail: jduan@nwsuaf.edu.cn

Supplementary Note 1

Synthetic procedures and spectroscopic data for compounds

All synthetic procedures and analytical data for compounds used in this study are detailed below.

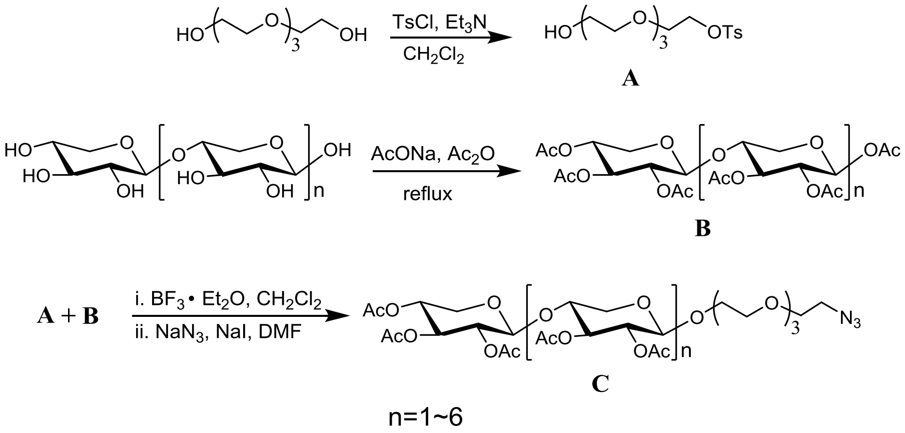

Supplementary Figure 1. Synthetic route of **C**.

Compound A: This compound was synthesized according to the previous reported procedure[1]. To a solution of tetraethylene glycol (10.19 g, 52.4 mmol) in CH_2Cl_2 (10 mL) was added tosyl chloride (1 g, 5.24 mmol) followed by TEA (1.1 mL, 0.796 g, 7.87 mmol) at 0 °C. The mixture was stirred at room temperature overnight, then poured into a separatory funnel and washed (3 × 10 mL) with water. The combined organic layers were dried (Na_2SO_4), filtered and concentrated in vacuo to afford compound **A** (1.73 g, 95%) as a colorless oil. ^{1}H NMR (500 MHz, $CDCl_3$), δ (ppm): 7.77 (d, J = 8.3 Hz, 2H), 7.32 (d, J = 8.0 Hz, 2H), 4.13 (d, J = 4.9 Hz, 2H), 3.73 – 3.52 (m, 14H), 2.42 (s, 3H).

Compound B: To a solution of Sodium acetate (100 mg, 1.22 mmol) in Acetic anhydride (1.4 mL) was added xylo-oligosaccharides (200 mg) at room temperature. After being heated under reflux for 1.5 h, the reaction solution was cooled to room temperature slowly and added CH_2Cl_2 (20 mL). The mix was washed successively with water (3 × 20 mL), $NaHCO_3$ (3 × 20 mL) and brine (3 × 20 mL). The organic phase was then dried (Na_2SO_4), filtered and concentrated in vacuo to afford compound **B** (210 mg) as a yellow oil. The successful synthesis of compound **B** was demonstrated by ^{1}H NMR spectra, ^{13}C NMR spectra and HRMS. In the ^{1}H NMR spectra of compound **B**, the typical signal peaks of Acetyl protons appeared at 1.8 – 2.2 ppm. The typical signal

peaks of skeletal protons were observed at 5.61 ppm, 5.27 ppm, 5.20 – 4.96 ppm, 4.92 ppm, 4.86 – 4.81 ppm, 4.73 ppm, 4.59 – 4.49 ppm, 4.45 ppm, 4.14 – 3.59 ppm, 3.62 ppm and 3.48 – 3.21 ppm. In the ^{13}C NMR spectra of compound **B**, the typical signal peaks of Acetyl carbons appeared at 169.92 ppm, 169.89 ppm, 169.77 ppm, 169.62 ppm, 169.56 ppm, 169.52 ppm, 169.30 ppm, 169.11 ppm, 168.96 ppm, 20.85 ppm, 20.74 ppm, 20.69 ppm, 20.61 ppm, 20.59 ppm and 20.56 ppm. The typical signal peaks of skeletal carbons appeared at 100.39 ppm, 99.89 ppm, 99.62 ppm, 99.46 ppm, 92.24 ppm, 92.17 ppm, 89.24 ppm, 75.05 ppm, 74.70 ppm, 74.62 ppm, 74.23 ppm, 72.01 ppm, 71.94 ppm, 70.97 ppm, 70.54 ppm, 70.47 ppm, 70.42 ppm, 70.37 ppm, 70.31 ppm, 69.90 ppm, 69.76 ppm, 69.50 ppm, 68.36 ppm, 68.28 ppm, 63.37 ppm, 62.60 ppm, 61.65 ppm and 61.50 ppm. In the HRMS of compound **B**, m/z calc'd for $C_{9n+13}H_{12n+18}O_{6n+9}Na$ (n=1~6) $[M+Na]^+$: 557.1473, 773.2103, 989.2733, 1205.3363, 1421.3993, 1637.4623, found 557.1469, 773.2105, 989.2726, 1205.3382, 1421.4021, 1637.4667. These results confirmed that the synthesis of compound **B** was successful.

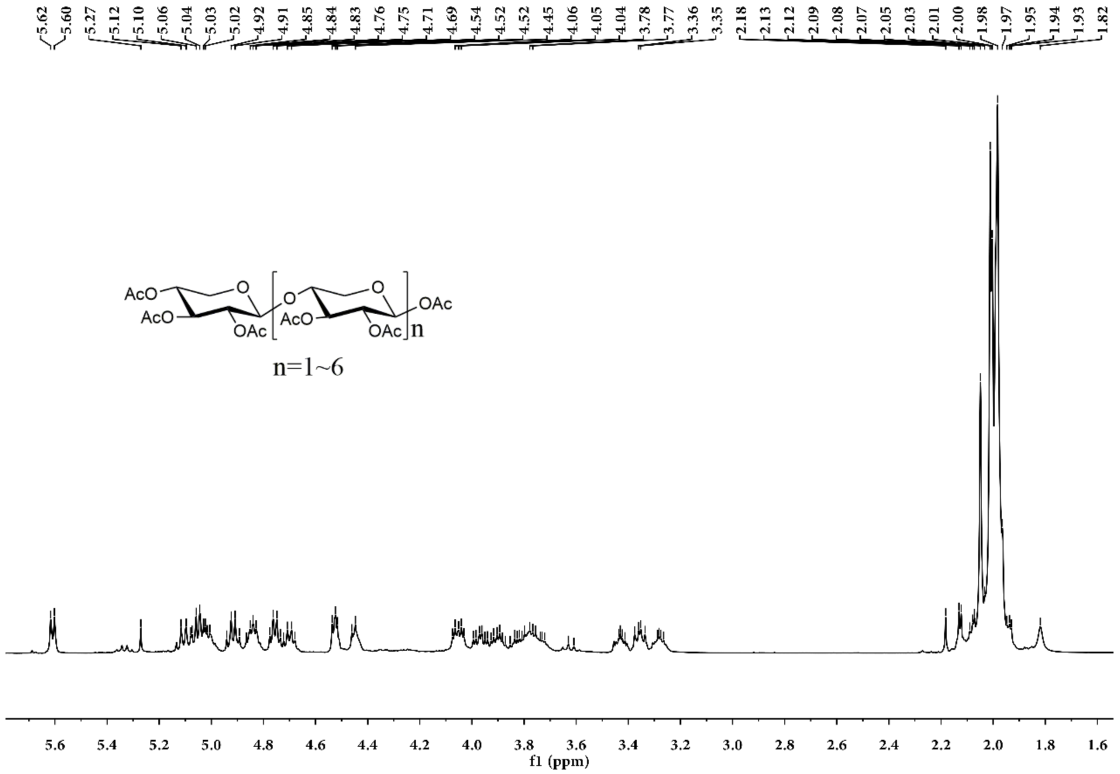

Supplementary Figure 2. ^{1}H NMR spectrum of Compound **B** in $CDCl_3$.

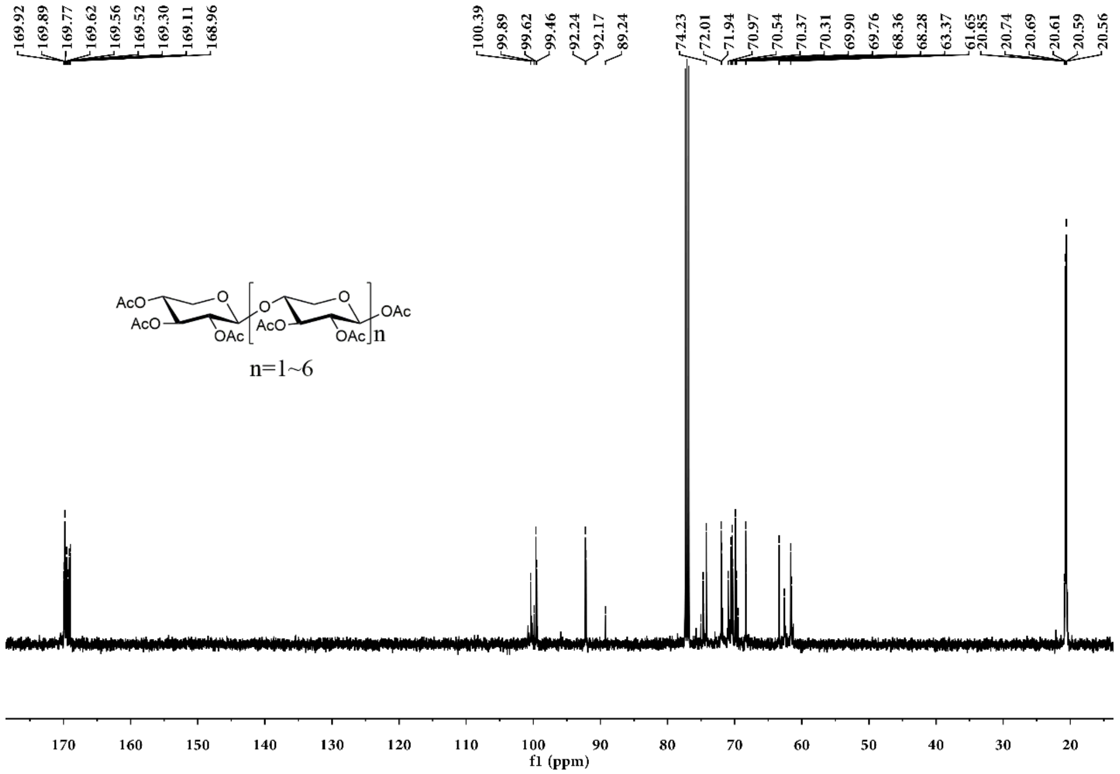

Supplementary Figure 3. ^{13}C NMR spectrum of Compound **B** in $CDCl_3$.

Compound C: To a stirring solution of compound **A** (388 mg, 1.1 mmol) and compound **B** (240 mg) in dry CH_2Cl_2 (4 mL) under a nitrogen atmosphere and at 0 °C was added dropwise $BF_3{\cdot}Et_2O$ (132 µL, 1.1 mmol) over a 15 min period. After stirring overnight at room temperature, the reaction solution was added CH_2Cl_2 (20 mL) and washed successively with $NaHCO_3$ (3 × 20 mL), water (3 × 20 mL) and brine (3 × 20 mL). The organic phase was then dried (Na_2SO_4), filtered and concentrated in vacuo to afford a yellow oil (380 mg). To a stirring solution of the yellow oil (380 mg) in dry DMF (8 mL) under a nitrogen atmosphere was added sodium azide (130 mg, 2 mmol). After stirring overnight (20 h) at 80 °C, the reaction solution was added CH_2Cl_2 (20 mL) and washed successively with water (3 × 20 mL), and brine (3 × 20 mL). The organic phase was then dried (Na_2SO_4), filtered and concentrated in vacuo. The residue was purified by column chromatography using EtOAc as eluent. The product **C** was isolated as a yellow oil (230 mg). The successful synthesis of compound **C** was demonstrated by ^{1}H NMR spectra, ^{13}C NMR spectra, FTIR spectra and HRMS. In the ^{1}H NMR spectra of compound **C**, the typical signal peaks of Glycol group protons appeared at 3.71 – 3.56 ppm. In the ^{13}C NMR spectra of compound **C**, the typical signal

peaks of Glycol group carbons appeared at 72.52 ppm, 70.69 ppm, 70.66 ppm, 70.59 ppm, 70.35 ppm, 70.03 ppm, 61.70 ppm. The typical signal peaks of the carbons of the CH_2N_3 appeared at 50.68 ppm. In the FTIR spectrum of compound **C**, the characteristic peak of azide group was observed at 2100 cm^{-1}. In the HRMS of compound **C**, m/z calc'd for $C_{9n+19}H_{12n+31}O_{6n+11}N_3Na$ (n = 1~6) $[M+Na]^+$: 716.2478, 932.3108, 1148.3738, 1364.4368, 1580.4998, 1796.5628, found 716.2486, 932.3110, 1148.3738, 1364.4360, 1580.4977, 1796.5584. These results confirmed that the synthesis of compound **C** was successful.

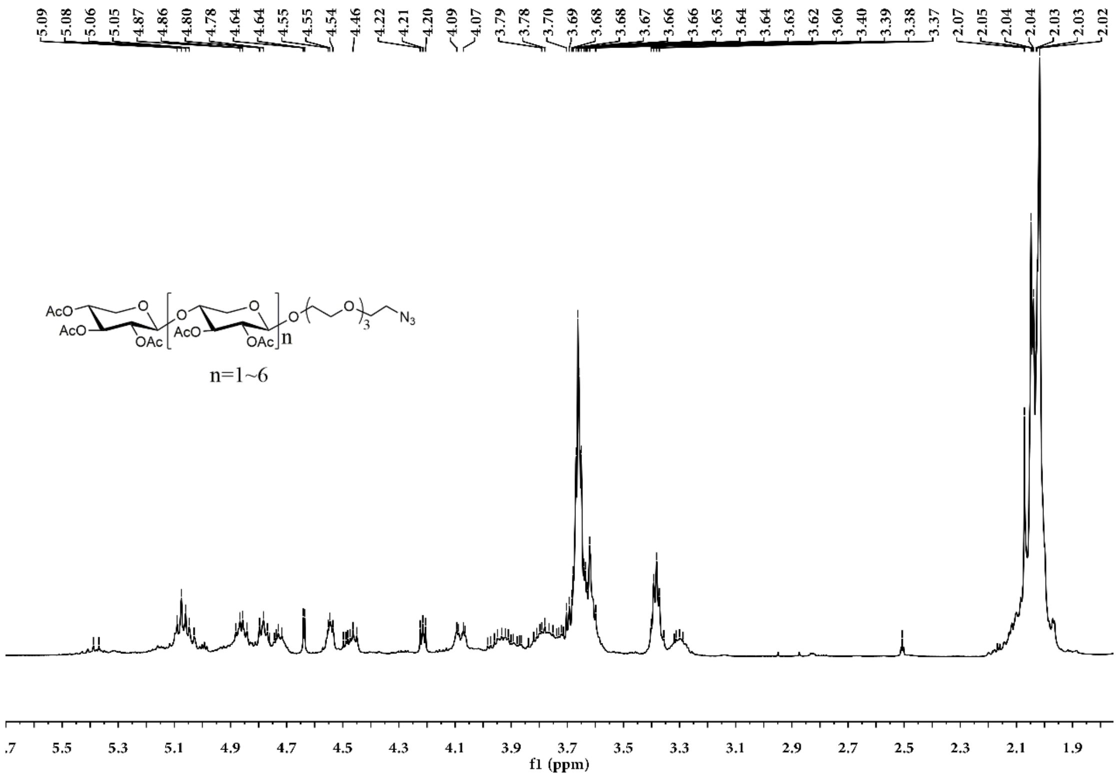

Supplementary Figure 4. ^{1}H NMR spectrum of Compound **C** in $CDCl_3$.

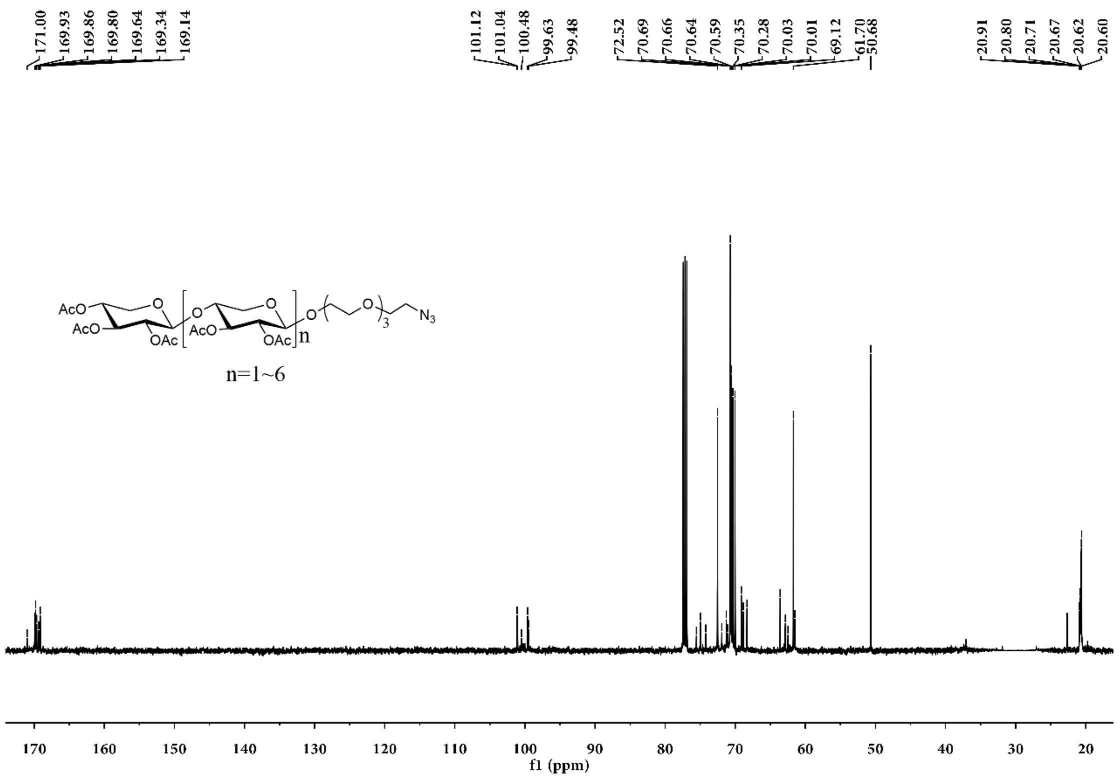

Supplementary Figure 5. ^{13}C NMR spectrum of Compound **C** in $CDCl_3$.

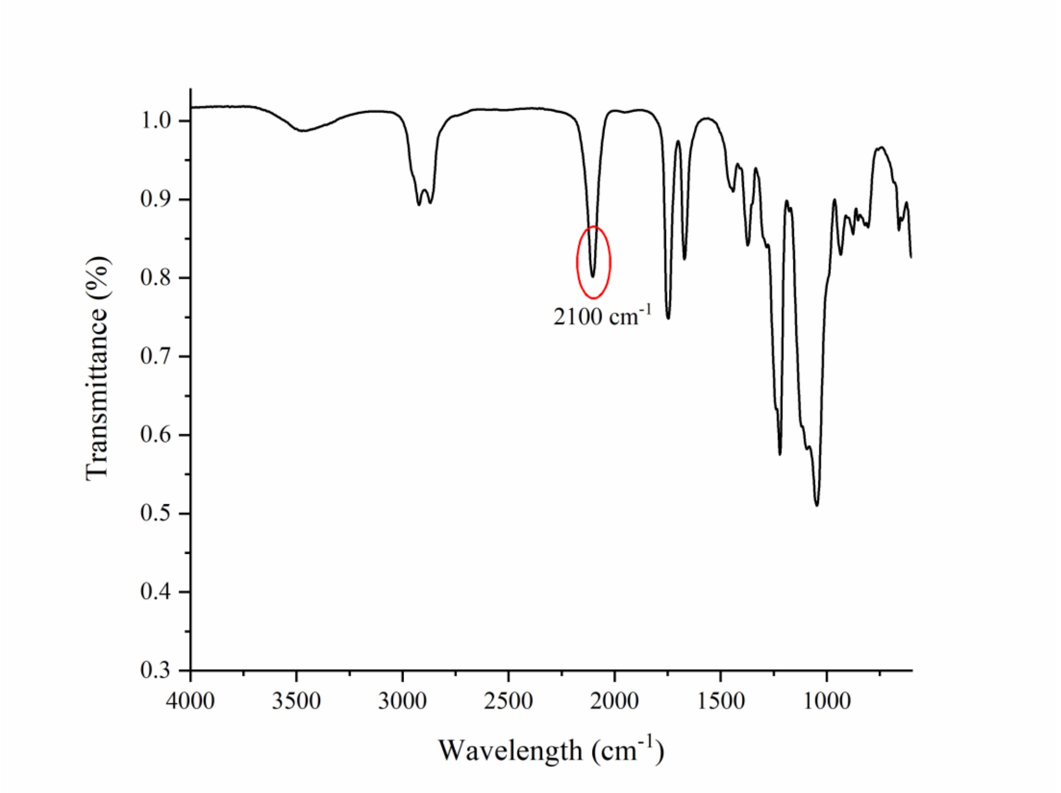

Supplementary Figure 6. FTIR spectra of Compound **C**.

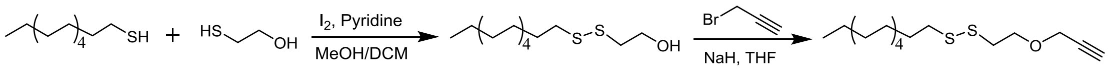

D **E**

Supplementary Figure 7. Synthetic route of **E**.

Compound D: This compound was synthesized according to the previous reported procedure[2]. To a stirring solution of dodecane-1-thiol (5.92 mL, 24.70 mmol) and 2-mercaptoethanol (1.739 mL, 24.70 mmol) DCM/MeOH (75:25, 250 mL) was added pyridine (4.29 mL, 49.4 mmol) followed by the gradual addition of diiodine (6.27 g, 24.70 mmol). The solution stirred for 5 h at room temperature, then the solvents were evaporated under reduced pressure and the resulting solid was dissolved in EtOAc and washed with brine. The organic layer was collected and dried over anhydrous sodium sulfate. Then, the mixture was filtered, concentrated, and purified by column chromatography using EtOAc/Petroleum ether (1:3, v/v) as eluent to afford the compound **D** (3.072 g, 11.03 mmol, 44.6%) as a waxy solid. ^{1}H NMR (500 MHz, $CDCl_3$), δ (ppm): 3.88 (t, J = 5.7 Hz, 2H), 2.84 (t, J = 5.8 Hz, 2H), 2.74 – 2.64 (m, 2H), 2.09 (s, 1H), 1.71 – 1.61 (m, 2H), 1.40 – 1.20 (m, 18H), 0.87 (t, J = 6.9 Hz, 3H).

Compound E: Compound **D** (2.9 g,10.4 mmol) and NaH (748.8 mg, 31.2 mmol) were suspended in dry THF (120 mL) and the suspension was stirred at 0 °C for 1 h. Propargyl bromide (2.48 g, 20.8 mmol) was added dropwise and the reaction mixture was stirred at room temperature overnight. After quenching with MeOH, CH_2Cl_2 (200 mL) was added to the reaction mixture. The organic layer was washed with H_2O, and then dried over Na_2SO_4. The filtrate was concentrated to yield the crude product, which was purified by column chromatography using CH_2Cl_2/Petroleum ether (1:3, v/v) as eluent. Product **E** was isolated as a yellow oil (1.96 g, 60.4 %). ^{1}H NMR (500 MHz, $CDCl_3$) , δ (ppm): 4.19 (d, J = 2.4 Hz, 2H), 3.79 (t, J = 6.6 Hz, 2H), 2.89 (t, J = 6.6 Hz, 2H), 2.74 –2.67 (m, 2H), 2.44 (t, J = 2.4 Hz, 1H), 1.71 – 1.63 (m, 2H), 1.38 – 1.22 (m, 18H), 0.88 (t, J = 6.9 Hz, 3H). ^{13}C NMR (126 MHz, $CDCl_3$), δ (ppm): 79.79, 75.02, 68.69, 58.55, 39.67, 38.62, 32.25, 29.98, 29.97, 29.93, 29.84, 29.68, 29.57, 29.52, 28.88, 23.02, 14.44. HRMS (ESI$^+$) m/z calc'd for $C_{17}H_{32}OS_2Na$ $[M+Na]^+$: 339.1785, found 339.1798

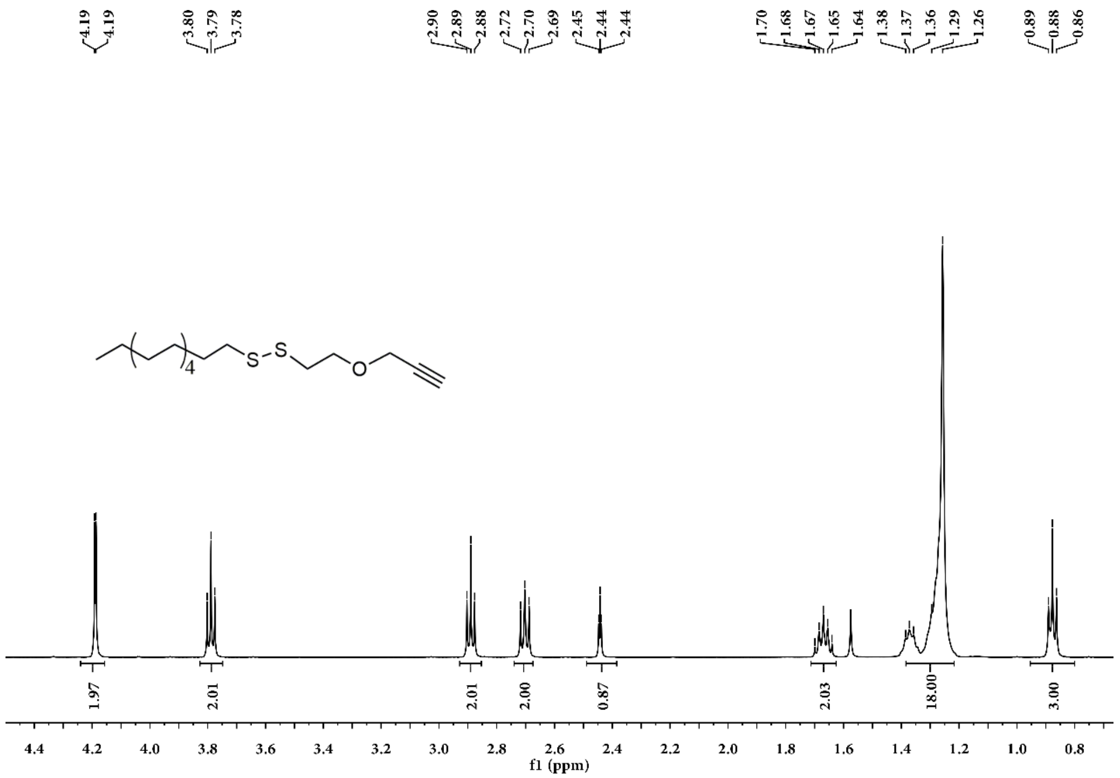

Supplementary Figure 8. ^{1}H NMR spectrum of Compound **E** in $CDCl_3$.

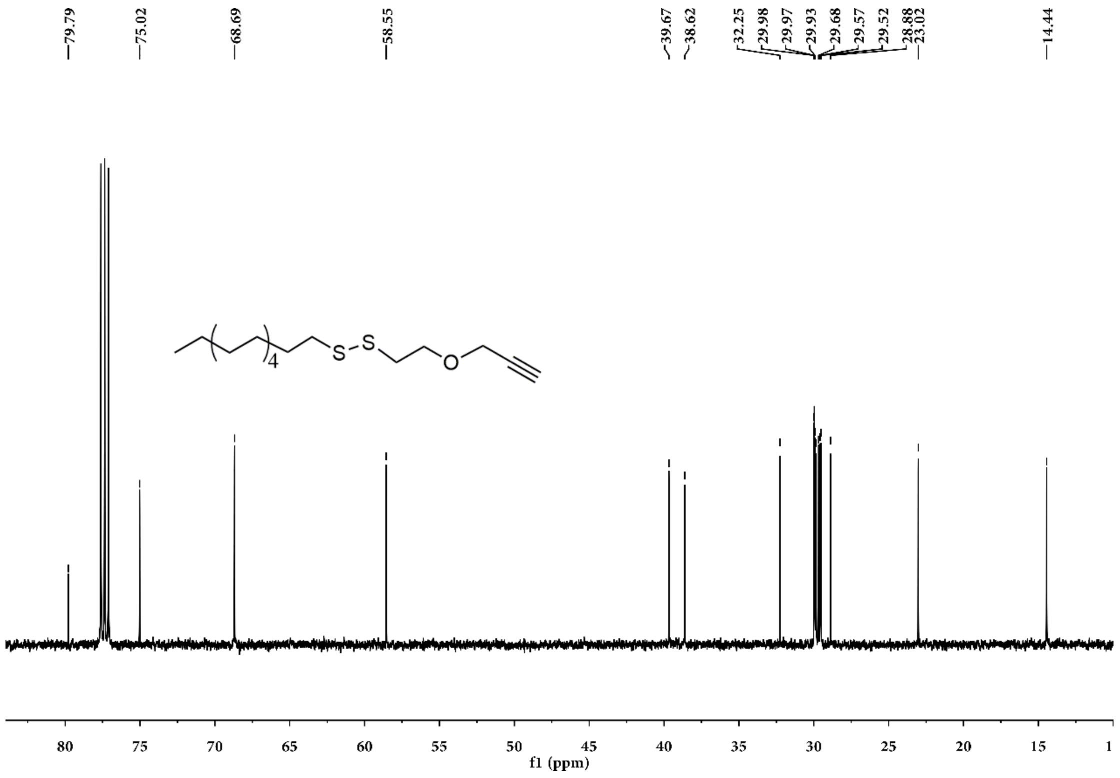

Supplementary Figure 9. ^{13}C NMR spectrum of Compound **E** in $CDCl_3$.

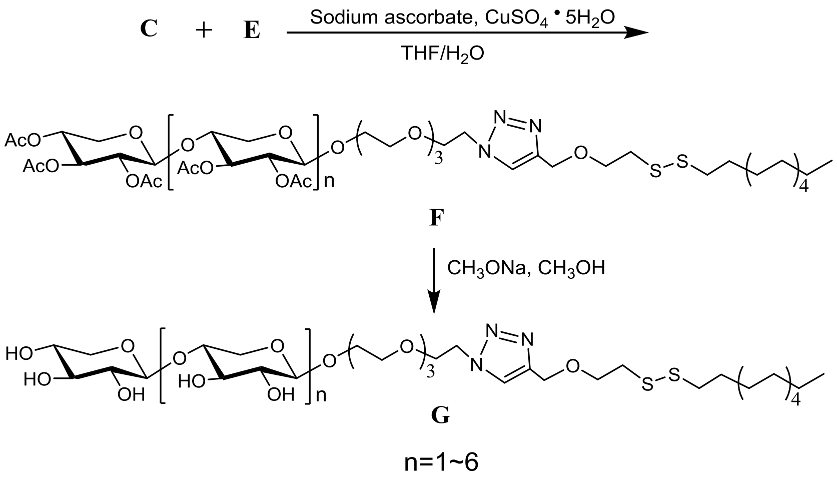

Supplementary Figure 10. Synthetic route of **G**.

Compound F: To a stirring solution of Compound **C** (200 mg) and Compound **E** (200 mg, 0.63mmol) in THF (5 mL) under a nitrogen atmosphere was added a solution of sodium ascorbate (11.5 mg, 0.058 mmol) and $CuSO_4 \cdot 5H_2O$ (7.25 mg, 0.029 mmol) in H_2O (2 mL). The reaction mixture was stirred at room temperature for 12 h. Then, the solvent was evaporated in vacuum. The residue was purified by column chromatography using EtOAc/Petroleum ether (3:1, v/v) as eluent. The product **F** was isolated as a yellow oil (210 mg). The successful synthesis of compound **F** was demonstrated by ^{1}H NMR spectra, ^{13}C NMR spectra and HRMS. In the ^{1}H NMR spectra of compound **F**, the typical signal peak of triazole ring proton appeared at 7.73 ppm. The typical signal peaks of alkyl protons were observed at 1.66 ppm, 1.30 ppm and 0.87 ppm. In the ^{13}C NMR spectra of compound **F**, the typical signal peaks of triazole ring carbons appeared at 144.64 ppm, 144.60 ppm, 123.72 ppm and 123.68 ppm. The typical signal peaks of alkyl carbons were observed at 39.10 ppm, 38.27 ppm, 31.79 ppm, 29.52 ppm, 29.50 ppm, 29.47 ppm, 29.39 ppm, 29.21 ppm, 29.12 ppm, 29.05 ppm, 28.41 ppm, 22.56 ppm, 14.02 ppm. In the HRMS of compound **F**, m/z calc'd for $C_{9n+36}H_{12n+63}O_{6n+12}S_2N_3Na$ (n=1~6) $[M+Na]^+$: 1032.4365, 1248.4995, 1464.5625, 1680.6255, 1896.6885, 2112.7515, found 1032.4335, 1248.4932, 1464.5525, 1680.6240, 1896.6878, 2112.7512. These results confirmed that the synthesis of compound **F** was successful.

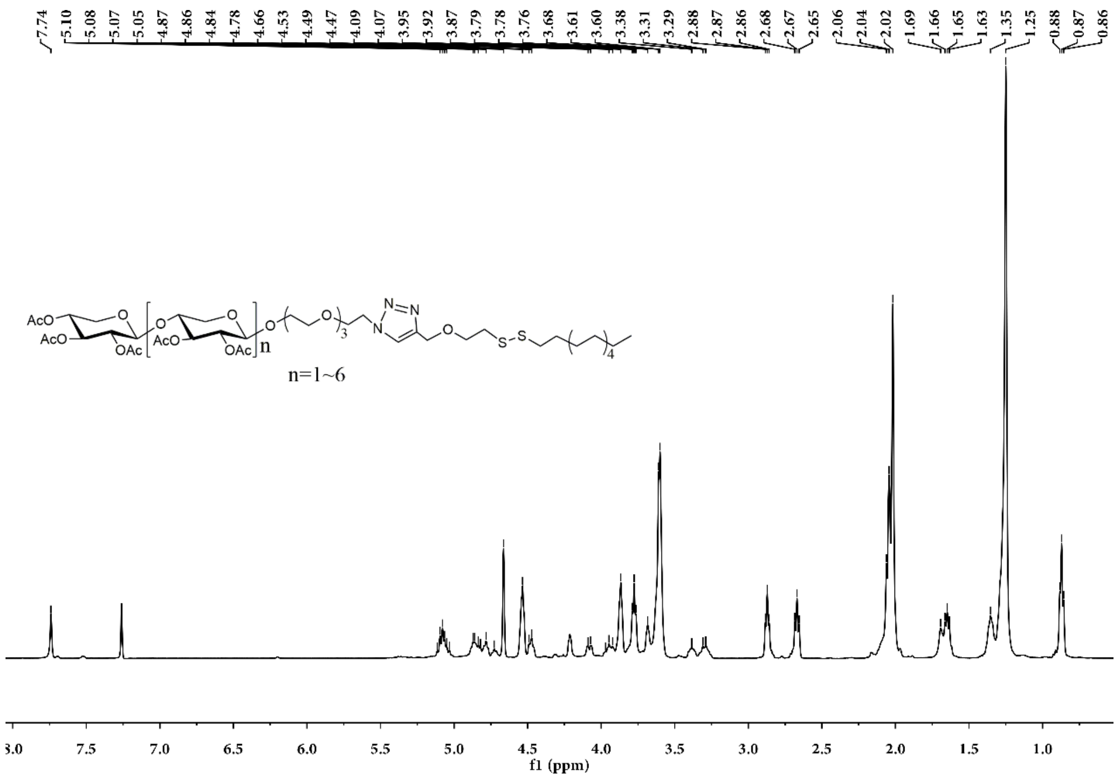

Supplementary Figure 11. ^{1}H NMR spectrum of Compound **F** in $CDCl_3$.

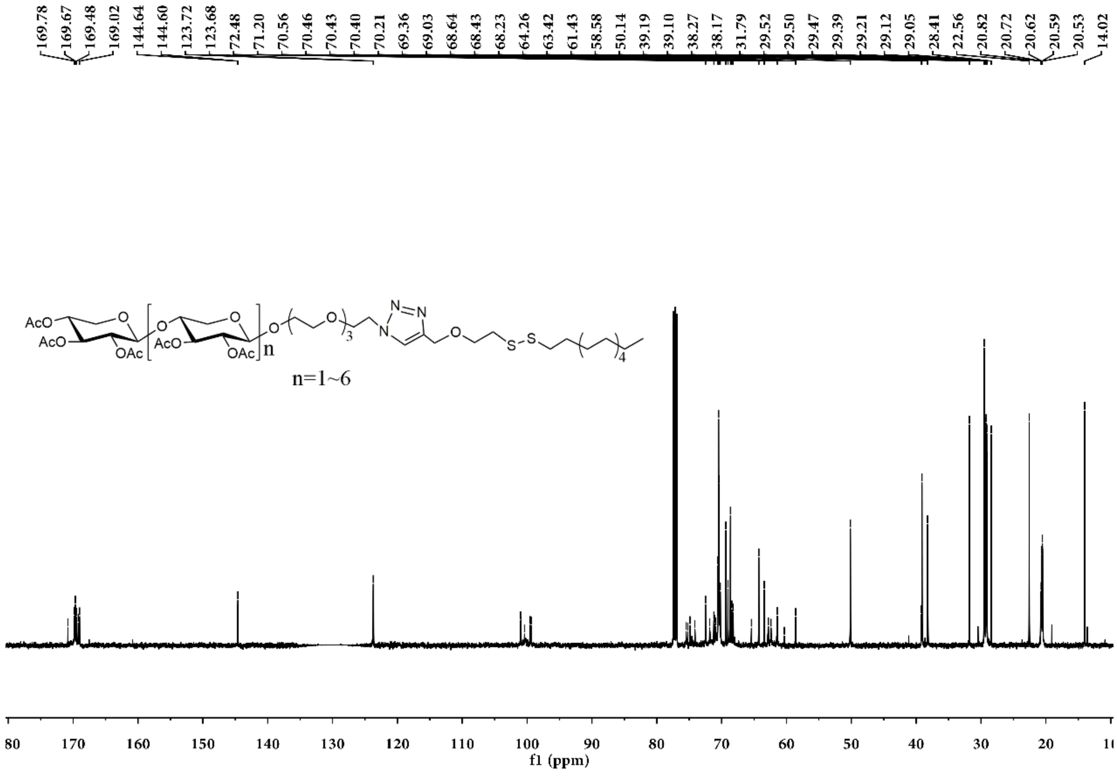

Supplementary Figure 12. ^{13}C NMR spectrum of Compound **F** in $CDCl_3$.

Compound **G**: To a solution of compound **F** (200 mg) in dry MeOH (5 mL), CH_3ONa

(10.8 mg, 0.2 mmol) was added. The reaction mixture was stirred at room temperature for 12 h. The solution was then neutralized by addition of ion-exchange resin (Amberlite IR 120 H^+) until pH = 7, filtered, and the solvent was removed under reduced pressure to give the final product of **G** as a yellow solid (150 mg). The successful synthesis of compound **G** was demonstrated by ^{1}H NMR spectra, ^{13}C NMR spectra and HRMS. In the ^{1}H NMR spectra of compound **G**, the signal peaks of acetyl protons disappeared at 1.9 – 2.2 ppm. In the ^{13}C NMR spectra of compound **G**, the signal peaks of acetyl carbons disappeared at 170.81 ppm, 169.78 ppm, 169.67 ppm, 169.48 ppm, 169.21 ppm, 169.02 ppm, 20.82 ppm, 20.72 ppm, 20.62 ppm, 20.59 ppm and 20.53 ppm. In the HRMS of compound **G**, m/z calc'd for $C_{5n+30}H_{8n+57}O_{4n+9}S_2N_3Na$ (n=1~6) $[M+Na]^+$: 822.3840, 954.4260, 1086.4680, 1218.5100, 1350.5520, 1482.5940, found 822.3854, 954.4273, 1086.4683, 1218.5092, 1350.5496, 1482.5892. These results confirmed that the synthesis of compound **G** was successful.

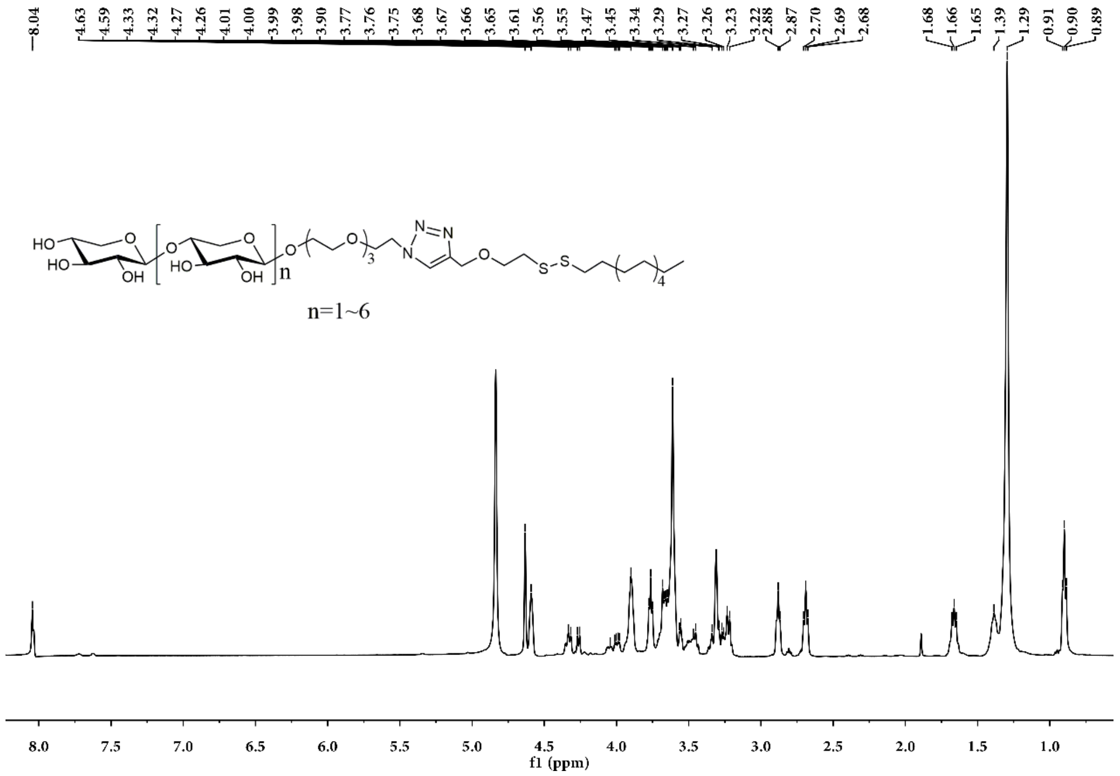

Supplementary Figure 13. ^{1}H NMR spectrum of Compound **G** in CD_3OD.

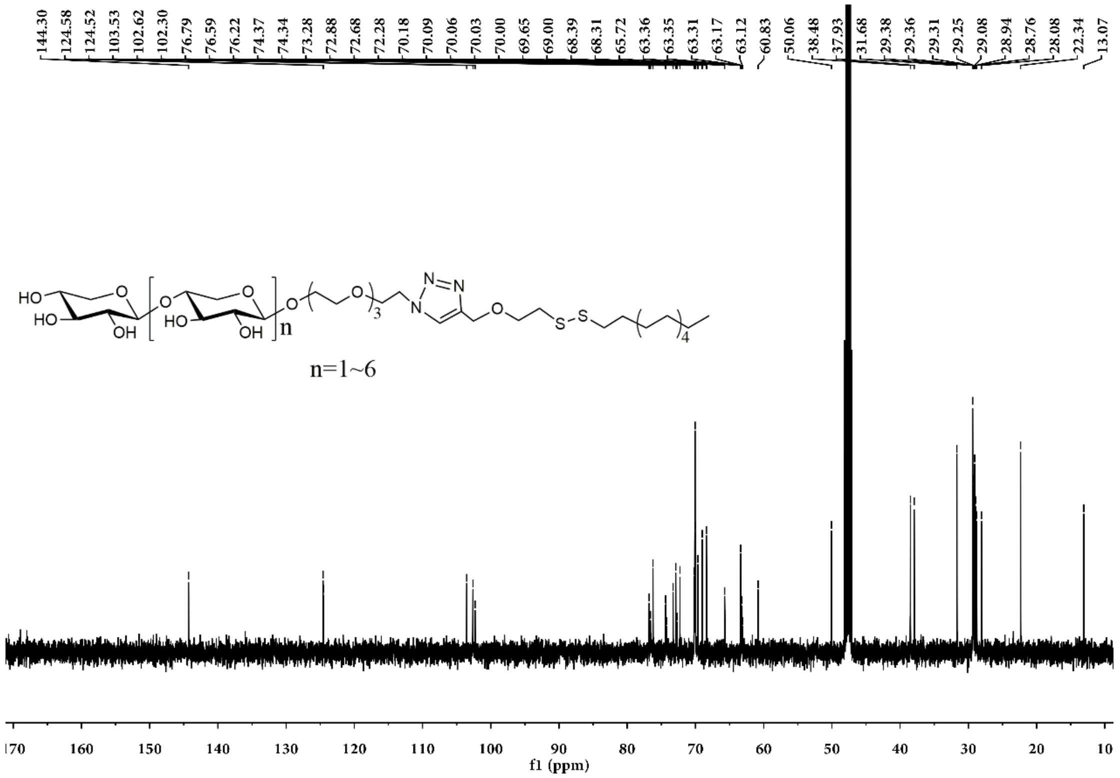

Supplementary Figure 14. ^{13}C NMR spectrum of Compound **G** in CD_3OD.

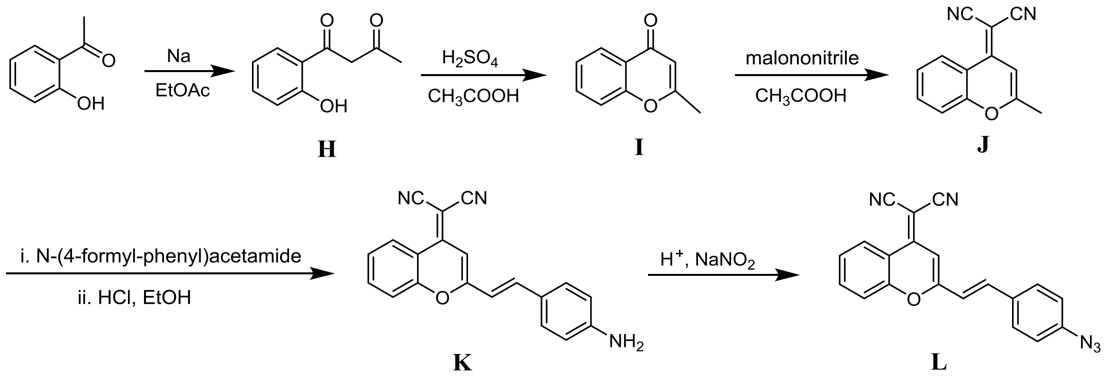

Supplementary Figure 15. Synthetic route of **L (Fluo)**.

Compound H: This compound was synthesized according to the previous reported procedure[3]. 1-(2-hydroxyphenyl)ethanone (10.0 g, 73.5 mmol) was dissolved in 200 mL ethyl acetate, and then sodium (8.00 g, 0.34 mmol) was added into the solution. The grayish-green solid was filtered after violently stirring for 4h at ambient temperature. The solid was dissolved in 100 mL deionized water, followed by the adjustment of pH of the solution to neutral. The aqueous solution was extracted with 200 mL EtOAc and the organic layers were dried over Na_2SO_4, filtered, and concentrated to yield the crude

product of compound **H** as a brown solid (7.34 g, 56%) which was directly used in the next reaction without further purification.

Compound I: This compound was synthesized according to the previous reported procedure[3]. Sulfuric acid (4.6 mL) was slowly added to a AcOH solution (70 mL) containing compound **H** (7.34 g, 41.1 mmol). The mixture was refluxed for about 30 min and then was poured into 800 mL ice water, followed by the adjustment of pH of the solution to neutral with Na_2CO_3. The aqueous solution was extracted with methylene dichloride twice and the organic layers were dried over Na_2SO_4, filtered, and concentrated to yield the crude product of compound **I** as an acicular gray solid (4.67 g, 71%). The crude product was directly used in the next reaction without further purification.

Compound J: This compound was synthesized according to the previous reported procedure[3]. Compound **I** (4.5 g, 28.1 mmol) and malononitrile (2.40 g, 36.2 mmol) were dissolved in 25 mL acetic anhydride. The solution was refluxed for 14 h and then the solvent was evaporated in vacuo. Deionized water (80 mL) was added to the residue and the mixture was refluxed for another 0.5 h, followed by extraction with methylene dichloride. The organic layers were dried over Na_2SO_4, filtered, and concentrated. The obtained crude product was purified by silica column chromatography to yield compound **J** as an orange solid (1.70 g, 29 %). ^{1}H NMR (500 MHz, $CDCl_3$), δ (ppm): 8.92 (d, 1H, J = 8.0 Hz), 7.72 (t, 1H, J = 8.0 Hz), 7.45 (t, 2H, J = 8.0 Hz), 6.72 (s, 1H), 2.44 (s, 3H).

Compound K: This compound was synthesized according to the previous reported procedure[3]. compound **J** (1 g, 4.75 mmol), and N-(4-formyl- phenyl)acetamide (710 mg, 4.4 mmol) were dissolved in toluene (50 mL) with piperidine (0.75 mL) and acetic acid (0.75 mL) under argon protection at room temperature. Then the mixture was refluxed for 3 h to give an orange precipitate. After filtration, the orange solid was refluxed in a solution of conc. HCl and ethanol (2:1, 150 mL) for another 2 h before the pH of the solution was adjusted to neutral. The aqueous solution was extracted with ethyl acetate and then the organic layers were dried over Na_2SO_4, filtered, and concentrated to obtain the crude product which was purified by silica column

chromatography to yield compound **K** as a crimson solid (610 mg, 41.3%). ^{1}H NMR (500 MHz, DMSO), δ (ppm): 8.73 (d, 1H, J = 8.0 Hz), 7.89 (t, 1H, J = 8.0 Hz), 7.77 (d, 1H, J = 8.0 Hz), 7.64 (d, 1H, J = 16.0 Hz), 7.58 (t, 1H, J = 8.0 Hz), 7.49 (d, 1H, J = 8.0 Hz), 7.08 (d, 1H, J = 16.0 Hz), 6.87 (s, 1H), 6.61 (d, 2H, J = 8.0 Hz), 5.99 (s, 2H).

Compound L: This compound was synthesized according to the previous reported procedure[3]. A solution of $NaNO_2$ (1.22 g, 17.69 mmol) in 30 mL of water was added dropwise to a solution of compound **K** (610 mg, 1.98 mmol) in 4 M HCl (80 mL) at 0-5 °C. After stirring the mixture at this temperature for 45 min, a solution of NaN_3 (2.75 g, 122 mmol) in water (30 mL) was added slowly to the mixture at the same temperature. Stirring was continued for 1 h below 5 °C and then at room temperature for another 1h. The yellow precipitate obtained was filtered and air-dried. The crude product was purified by silica gel column chromatography to yield compound **L** as a yellow solid (290.3 mg, 43.5%). ^{1}H NMR (500 MHz, Acetone-d_6), δ (ppm): 8.87 (d, 1H, J = 8.0 Hz), 7.93 (t, 1H, J = 8.0 Hz), 7.85 (d, 2H, J = 8.0 Hz),7.84 (d, 1H, J = 16.0 Hz), 7.77 (d, 1H, J = 8.0 Hz), 7.62 (t, 1H, J = 8.0 Hz), 7.41 (d, 1H, J = 16.0 Hz), 7.20 (d, 2H, J = 8.0 Hz), 6.99 (s, 1H).

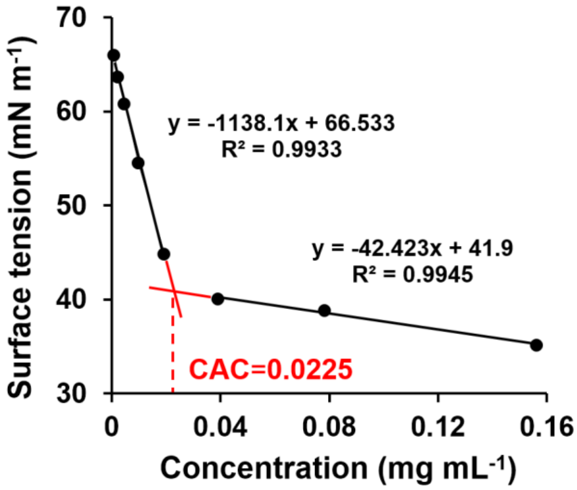

Supplementary Figure 16. A plot of the surface tension of water *vs.* the concentration of AM. The crossing point implies that the CAC is approximately 0.0225 mg mL^{-1}.

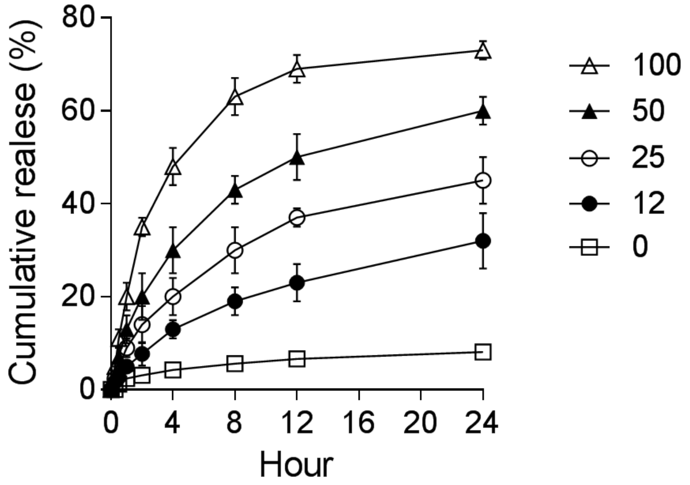

Supplementary Figure 17. CIP release from AM-CIP incubated in PBS at 37°C with different amounts of Na_2S (0 – 100 μM).

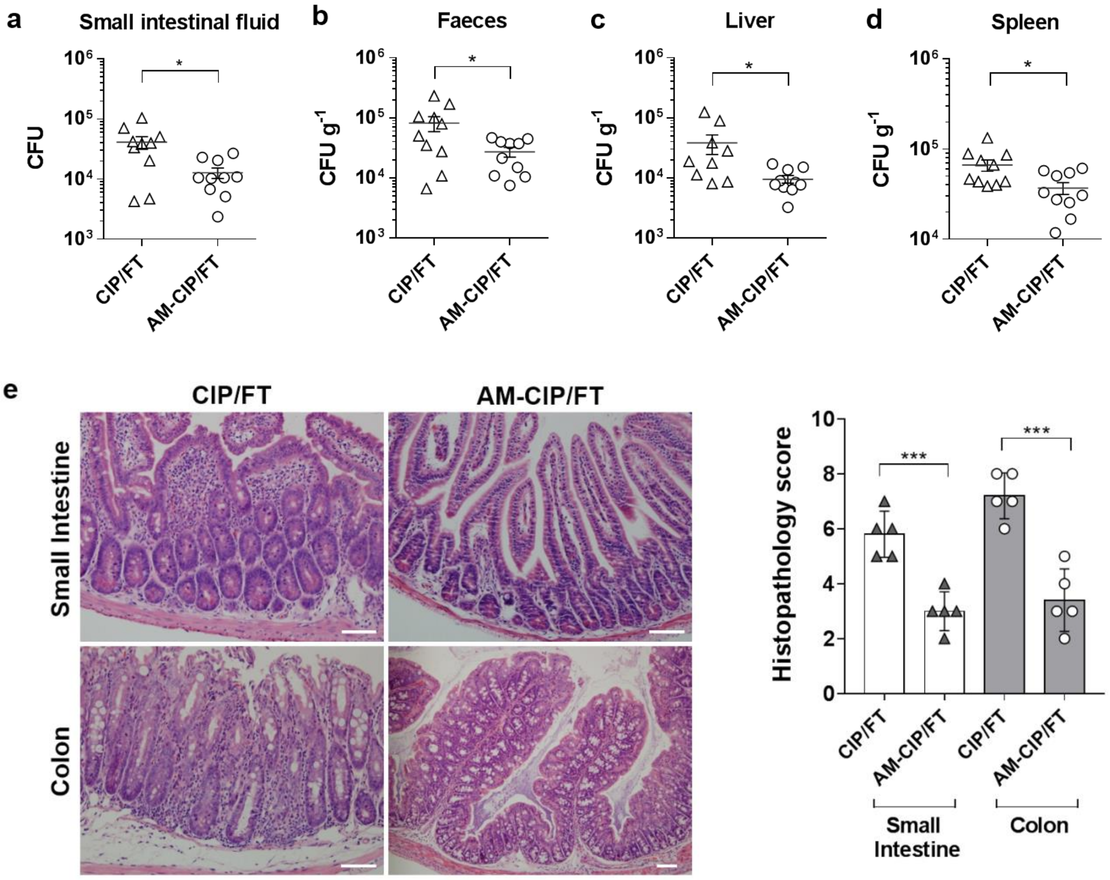

Supplementary Figure 18. Faecal transplantation (FT) from AM-CIP-treated infection model to infected mice ameliorates symptoms of acute intestinal infection. Infected mice were colonized with faeces for 6 days from infection model mice treated with CIP or AM-CIP, followed by measurement of bacterial burden in the small intestine (**a**), faeces (**b**), liver (**c**) and spleen (**d**). n = 9 – 10 per group. Data are presented as mean ± SEM. *$P < 0.05$. (**e**) Representative H&E stained intestine tissues. Scale bar, 50 µm. Histogram represents combined histopathology score. Data are shown as mean ± SEM, and each dot represents one animal, n = 5. ***$P < 0.001$.

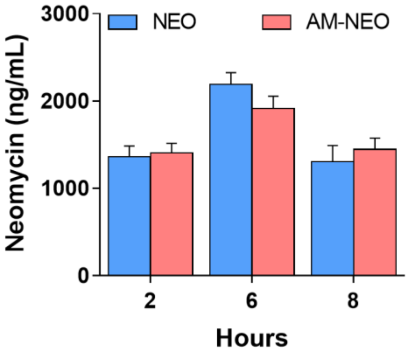

Supplementary Figure 19. Mean intestinal concentration of neomycin (NEO) following a single oral administration of NEO (10 mg kg^{-1}) or equivalent AM-NEO in normal mice (n = 3 per group). The NEO concentration was determined by ELISA kit (REAGEN, Moorestown, USA). Data are shown as mean ± SD.

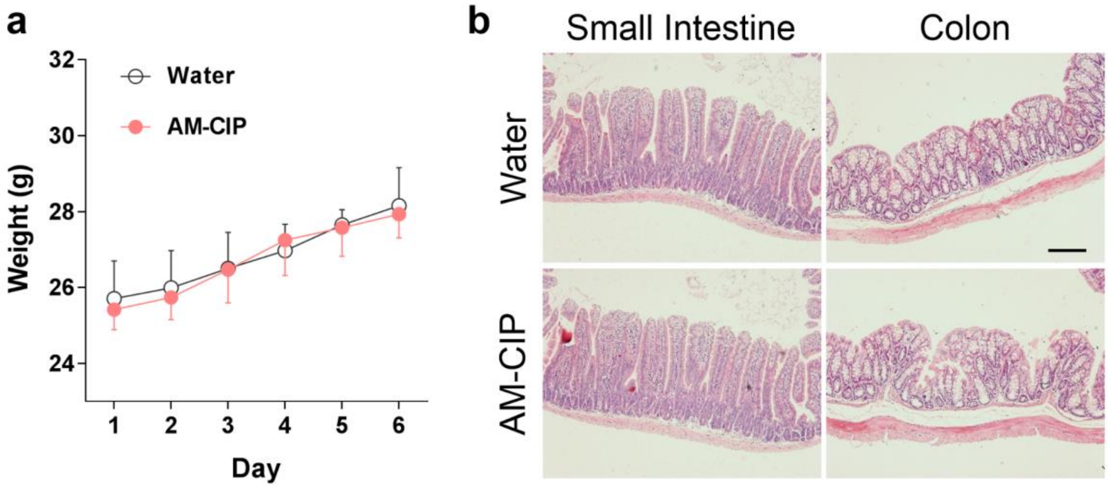

Supplementary Figure 20. *In vivo* toxicity evaluation of AM vesicles on normal mice. (**a**) Mice body weight log over 6 days. Error bars represent the SD of the mean. n = 5 mice per group. (**b**) H&E sections of intestine tissues isolated from mice on day 6. No apparent intestine mucosal epithelial morphology change or inflammation, suggesting that the AM-CIP are biocompatible and safe for oral administration in mouse models. Scale bar, 100 μm.

Supplementary References

1 Chirkin, E. *et al.* Neutralization of Pathogenic Fungi with Small-Molecule Immunotherapeutics. *Angewandte Chemie* **129**, 13216-13220 (2017).

2 Giesler, K. E., Marengo, J. & Liotta, D. C. Reduction Sensitive Lipid Conjugates of Tenofovir: Synthesis, Stability, and Antiviral Activity. *Journal of Medicinal Chemistry* **59**, 7097-7110 (2016).

3 Sun, W. *et al.* A two-photon fluorescent probe with near-infrared emission for hydrogen sulfide imaging in biosystems. *Chemical communications* **49**, 3890-3892 (2013).
